# Allograft Rejection and the Latent HIV Reservoir in Kidney Transplant Recipients With HIV

**DOI:** 10.1093/infdis/jiag154

**Published:** 2026-03-13

**Authors:** Andrew Sulaiman, Miruthula Tamil Selvan, Ping Yang, Xianming Zhu, Yolanda Eby, Sarah E Benner, Reinaldo E Fernandez, Sarah Hussain, Diane Brown, Niraj Desai, Sander Florman, Meenakshi M Rana, Rachel Friedman-Moraco, Marcus R Pereira, Shikha Mehta, Peter Stock, Alexander Gilbert, Michele I Morris, Valentina Stosor, Sapna A Mehta, Catherine B Small, Karthik Ranganna, Carlos A Q Santos, Saima Aslam, Maricar Malinis, Nahel Elias, Emily A Blumberg, Allan Massie, Melissa L Smith, Megan Morsheimer, Gregory M Laird, Robert Siliciano, Dorry L Segev, Christine M Durand, Andrew D Redd, Aaron A R Tobian

**Affiliations:** Department of Pathology, Johns Hopkins School of Medicine, Baltimore, Maryland, USA; Department of Pathology, Johns Hopkins School of Medicine, Baltimore, Maryland, USA; Department of Pathology, Johns Hopkins School of Medicine, Baltimore, Maryland, USA; Department of Pathology, Johns Hopkins School of Medicine, Baltimore, Maryland, USA; Department of Epidemiology, Johns Hopkins Bloomberg School of Public Health, Baltimore, Maryland, USA; Department of Pathology, Johns Hopkins School of Medicine, Baltimore, Maryland, USA; Department of Pathology, Johns Hopkins School of Medicine, Baltimore, Maryland, USA; Department of Medicine, Division of Infectious Diseases, Johns Hopkins School of Medicine, Baltimore, Maryland, USA; Department of Medicine, Division of Infectious Diseases, Johns Hopkins School of Medicine, Baltimore, Maryland, USA; Department of Medicine, Division of Infectious Diseases, Johns Hopkins School of Medicine, Baltimore, Maryland, USA; Department of Surgery, Johns Hopkins University School of Medicine, Baltimore, Maryland, USA; Recanati/Miller Transplantation Institute, Icahn School of Medicine at Mount Sinai, New York, New York, USA; Department of Medicine, Icahn School of Medicine at Mount Sinai, New York, New York, USA; Department of Medicine, Emory University, Atlanta, Georgia, USA; Department of Medicine, Columbia University Irving Medical Center, New York, New York, USA; Department of Medicine, University of Alabama Heersink School of Medicine, Birmingham, Alabama, USA; Department of Surgery, University of California, SanFrancisco, San Francisco, California, USA; Medstar Transplant Institute, Georgetown University School of Medicine, Washington, DC, USA; Department of Medicine, Division of Infectious Diseases, University of Miami Miller School of Medicine, Miami, Florida, USA; Departments of Medicine and Surgery, Divisions of Infectious Diseases and Organ Transplantation, Feinberg School of Medicine, Northwestern University, Chicago, Illinois, USA; Department of Medicine, New York University Grossman School of Medicine Langone Transplant Institute, New York, New York, USA; Department of Medicine, Division of Infectious Diseases, Weill Cornell Medicine, New York, New York, USA; Department of Medicine, Penn Transplant Institute, University of Pennsylvania, Philadelphia, Pennsylvania, USA; Department of Medicine, Drexel University, Philadelphia, Pennsylvania, USA; Division of Infectious Diseases, Rush University Medical Center, Chicago, Illinois, USA; Department of Medicine, University of California, San Diego, San Diego, California, USA; Department of Internal Medicine, Yale University School of Medicine, New Haven, Connecticut, USA; Department of Surgery and Transplant Center, Massachusetts General Hospital and Harvard Medical School, Boston, Massachusetts, USA; Department of Medicine, Perelman School of Medicine at the University of Pennsylvania, Philadelphia, Pennsylvania, USA; Department of Surgery, New York University Grossman School of Medicine, New York, New York, USA; Department of Biochemistry and Molecular Genetics, University of Louisville, Louisville, Kentucky, USA; National Institute of Allergy and Infectious Diseases, National Institutes of Health, Bethesda, Maryland, USA; Accelevir Diagnostics, Baltimore, Maryland, USA; Department of Medicine, Division of Infectious Diseases, Johns Hopkins School of Medicine, Baltimore, Maryland, USA; Department of Surgery, New York University Grossman School of Medicine, New York, New York, USA; Department of Medicine, Division of Infectious Diseases, Johns Hopkins School of Medicine, Baltimore, Maryland, USA; Department of Medicine, Division of Infectious Diseases, Johns Hopkins School of Medicine, Baltimore, Maryland, USA; National Institute of Allergy and Infectious Diseases, National Institutes of Health, Bethesda, Maryland, USA; Department of Pathology, Johns Hopkins School of Medicine, Baltimore, Maryland, USA; Department of Epidemiology, Johns Hopkins Bloomberg School of Public Health, Baltimore, Maryland, USA

**Keywords:** HIV, latent viral reservoir, HIV organ transplant

## Abstract

People with HIV-1 have higher risk for rejection after kidney transplantation but the mechanism is poorly understood. As HIV latency promotes immune dysregulation and chronic inflammation, we evaluated whether the size of the HIV latent viral reservoir (LVR) at baseline and through 52-weeks is associated with rejection in kidney transplant recipients with HIV from donors with and without HIV. Using the intact proviral DNA assay, we found no differences in the LVR between those who experienced rejection (n = 14) versus those who did not (N = 55) regardless of donor HIV status. These data support the feasibility of HIV+ to HIV+ organ transplantation.

***Clinical Trials Registration.*** NCT03500315.

Antiretroviral therapy (ART) effectively suppresses HIV-1 replication, reducing viral RNA in blood to below detectable thresholds and preventing viral rebound in people with HIV (PWH). However, ART is not curative. A major barrier to HIV cure, which to date has largely been achieved in select stem cell transplantation cases, is the presence of the latent viral reservoir (LVR), established early in HIV infection when activated CD4+ T cells become infected with an HIV provirus, and subsequently transitions to a long-lived resting state capable of reconstituting viremia if reactivated in the absence of ART [1–3]. The size of the LVR decreases slowly over the first 7 years of ART, but then largely persists at roughly constant levels longitudinally [4].

Kidney transplantation has been shown in multiple studies to be safe and effective for PWH and end-stage kidney disease. However, many studies have shown that PWH have a higher risk of kidney allograft rejection than those without HIV, with 1 year rejection risk between 25% and 50% [5–7]. The reason for this elevated risk is incompletely understood, and may be due to immune dysregulation. More recently, it has been shown that kidney transplantation into recipients with HIV from donors with HIV (HIV D+/R+) is safe and noninferior to donors without HIV (HIV D−/R+) [6–8]. In a pilot study, rejection was higher among those with HIV D+/R+ versus D−/R+, but this difference was not seen in the larger noninferiority study.

It is unknown whether the size and make-up of the LVR are also associated with allograft rejection [5]. We hypothesize that a larger or more immunologically active LVR, potentially influenced by donor HIV status may increase the risk of kidney allograft rejection. To better understand possible mechanisms of rejection in recipients with HIV, we sought to evaluate the LVR longitudinally using the intact proviral DNA assay (IPDA) which can quantify genetically intact proviruses (potentially replication competent) and defective (nonfunctional) proviruses for an accurate determination of the LVR.

## METHODS

### Study Population and Procedures

The participants in this study were part of the HIV Organ Policy Equity (HOPE) in Action kidney transplantation study, which has been described in detail previously (ClinicalTrials.gov number, NCT03500315) [6, 7, 9]. Briefly, kidney transplant recipients with HIV received a kidney from a deceased donor either with or without HIV. Peripheral blood mononuclear cells (PBMCs) were collected at the time of transplant (week 0) before the start of immunosuppression induction, and at subsequent post-transplant time points, including weeks 13, 26, and 52. Participants with baseline (week 0) and at least two follow-up time points (weeks 13, 26, and 52 post-transplantation) were included in this study. Rejection was defined as being clinically suspected and either treated rejection or biopsy-proven rejection according to Banff classification; all biopsies were reviewed centrally.

The recipient HOPE study was reviewed and approved by Johns Hopkins University School of Medicine Institutional Review Board (IRB00141138) centrally and by each local transplant center. All recipients provided written informed consent.

### Laboratory Testing

The IPDA was performed by Accelevir diagnostics [10]. Briefly, CD4+ T cells were isolated from PBMCs through immunomagnetic selection using EasySep human CD4+ T cell enrichment kit (Stemcell Technologies) after which genomic DNA was isolated through DNeasy Blood and Tissue Kit (Qiagen). IPDA was performed through duplex droplet digital PCR, which assesses specific regions in the HIV genome. These regions are the rev response element amplicon in the *env* gene that is highlighted by fluorescence in the VIC (2′-chloro-7′phenyl-1,4-dichloro-6-carboxyfluorescein) channel and the packaging signal, which is highlighted by fluorescence in FAM (6-carboxyfluorescein) channel. Based on the combination of VIC and FAM fluorescence, it is possible to estimate the status of the provirus (FAM+/VIC− indicates 3′ defective provirus, FAM−/VIC+ indicates 5′ defective provirus, and FAM+/VIC+ indicates intact provirus).

### Statistical Analysis

Baseline characteristics of the study population were summarized and stratified by kidney rejection status using descriptive statistics. Continuous variables were presented as medians with interquartile ranges (IQRs), and categorical variables as frequencies and percentages. Our primary variables were LVR biomarkers, including intact provirus, defective provirus, and the ratio of intact/defective provirus frequencies and the main outcome was rejection [11, 12]. All values were measured per million CD4+ T cells and log10 transformed for analysis. The size of LVR was compared between participants with and without kidney rejection, stratified by donor HIV status, and at weeks 0, 13, 26, and 52 after transplant using Wilcoxon's rank-sum tests.

## RESULTS

### Baseline Characteristics of Study Population

A total of 69 kidney transplant recipients who had baseline and at least two follow-up visits at weeks 13, 26, and 52 (median number of visits = 3) were included in this study (Supplementary Table 1). Of the 69 participants, 14 (20%) experienced allograft rejection. Baseline characteristics were compared between recipients with and without rejection. Recipients with rejection were younger at the time of transplant compared with those without rejection (median age 47 years [IQR 36–53] vs 54 years [IQR 49–63], *P* = .006). There was no significant difference in sex distribution. Among those with rejection, the median CD4+ T-cell count was 558 (IQR: 321 to 755) cells/µL, and 100% had an HIV viral load of <200 copies/mL. These values were not significantly different from participants without rejection, whose median CD4+ T-cell count was 498 (IQR: 382 to 736) cells/µL, and 98% had an HIV viral load of <200 copies/mL. Among the 55 participants who did not experience allograft rejection, 33 (60%) received a D+ organ. Among the 14 participants who experienced allograft rejection, half (7/14) received a transplant D+ organ. These 14 recipients experienced a total of 19 rejection episodes, 15 of which were biopsy-proven and all cellular-mediated. Treatment was administered for 16 episodes, most commonly with steroids (n = 11) (Supplementary Table 2).

### Longitudinal Trajectories in the Latent Viral Reservoir

**Figure 1.**
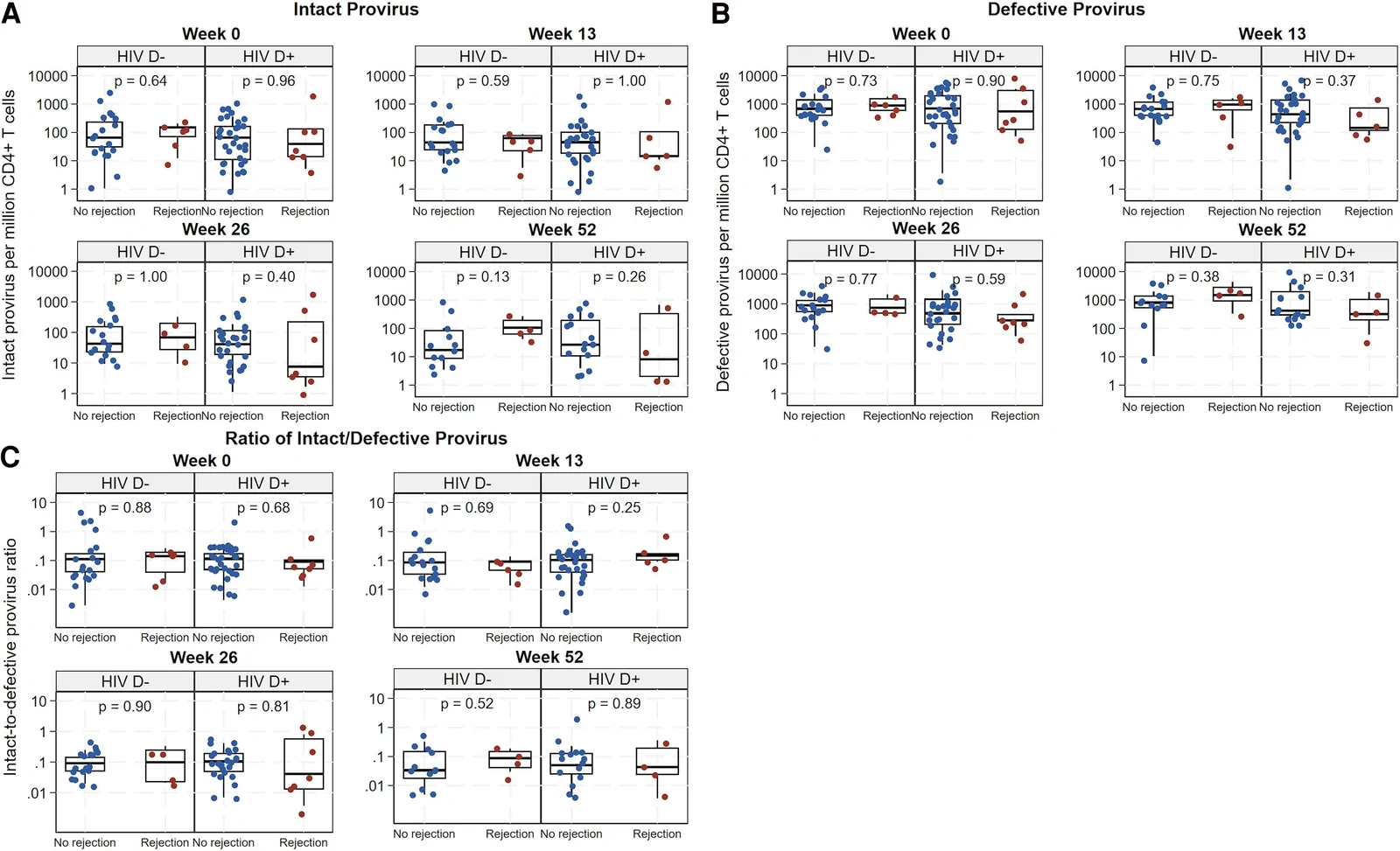
Intact and defective provirus frequencies per million CD4+ T cells by donor HIV status and allograft rejection. *A*, Intact (*B*) defective provirus per million CD4+ T cells (*C*) Intact-to-defective proviral ratios. Count per million CD4+ T cells is plotted on the y-axis using a log10 scale. Analyses were conducted longitudinally in weeks 0, 13, 26, and 52. Wilcoxon rank-sum tests were used to compare recipients with and without rejection at each time point stratified by donor HIV status. Among 14 recipients with kidney organ rejection, one was omitted from analysis due to missing provirus data. Because the y-axis is on a log10 scale, values within the same order of magnitude are plotted closely together, causing some data points to overlap. Abbreviations: HIV D+, donors with HIV; HIV D−, donors without HIV.

There were no significant differences in intact or defective proviruses, or the ratio of intact/defective provirus at any time point from baseline to 52 weeks post-transplant between participants with and without kidney rejection, regardless of donor HIV status (Figure 1*A*–*C*). Among D− recipients without rejection, intact provirus ranged from a median of 65.68 copies/10^6^ cells (IQR = 29.92–238.27) at baseline to 17.23 copies/10^6^ cells (IQR = 8.46–86.37) at week 52. Defective provirus ranged from a median of 683.19 copies/10^6^ cells (IQR = 381.8–1460.05) at baseline to 808.03 copies/10^6^ cells (IQR = 515.04–1433.14) at week 52. The ratio of intact-to-defective provirus ranged from a median of 0.111 copies/10^6^ cells (IQR = 0.040–0.181) to 0.034 copies/10^6^ cells (IQR = 0.017–0.155) over the same period. Similarly, in D− recipients with rejection, intact provirus ranged from a median of 150.58 copies/10^6^ cells (IQR = 68.38–156.72) at baseline to 104.36 copies/10^6^ cells (IQR = 59.82–200.00) at week 52. Defective provirus counts ranged from a median of 878.05 copies/10^6^ cells (IQR = 581.74–1599.70) at baseline to 1497.80 copies/10^6^ cells (IQR = 890.33–2934.36) week 52, and the ratio of intact/defective provirus ranged from a median of 0.141 copies/10^6^ cells (IQR = 0.038–0.200) to 0.088 copies/10^6^ cells (IQR = 0.040–0.156) over the same period.

Among HIV D+/R+ participants without rejection, intact provirus ranged from a median of 65.10 copies/10^6^ cells (IQR = 10.79–171.84) at baseline to 26.95 copies/10^6^ cells (IQR = 10.21–202.97) at week 52. Between baseline and week 52, defective provirus ranged from a median of 706.34 copies/10^6^ cells (IQR = 196.34–2060.71) to 417.63 copies/10^6^ cells (IQR = 287.82–2061.27), and the ratio of intact/defective provirus ranged from a median of 0.114 copies/10^6^ cells (IQR = 0.047–0.180) to 0.050 copies/10^6^ cells (IQR = 0.024–0.131). Similarly, in participants who received a D+ organ and who experience rejection, intact provirus ranged from a median of 39.17 copies/10^6^ cells (IQR = 13.38–136.77) at baseline to 8.18 copies/10^6^ cells (IQR = 1.93–341.74) at week 52. Defective provirus ranged from a median of 547.39 copies/10^6^ cells (IQR = 121.44–3190.24) at baseline to 323.01 copies/10^6^ cells (IQR = 189.47–1103.28) week 52, and the ratio of intact/defective provirus ranged from a median of 0.091 copies/10^6^ cells (IQR = 0.050–0.110) to 0.044 copies/10^6^ cells (IQR = 0.023–0.200) over the same period.

### Changes in the Latent Viral Reservoir After Allograft Rejection

**Figure 2.**
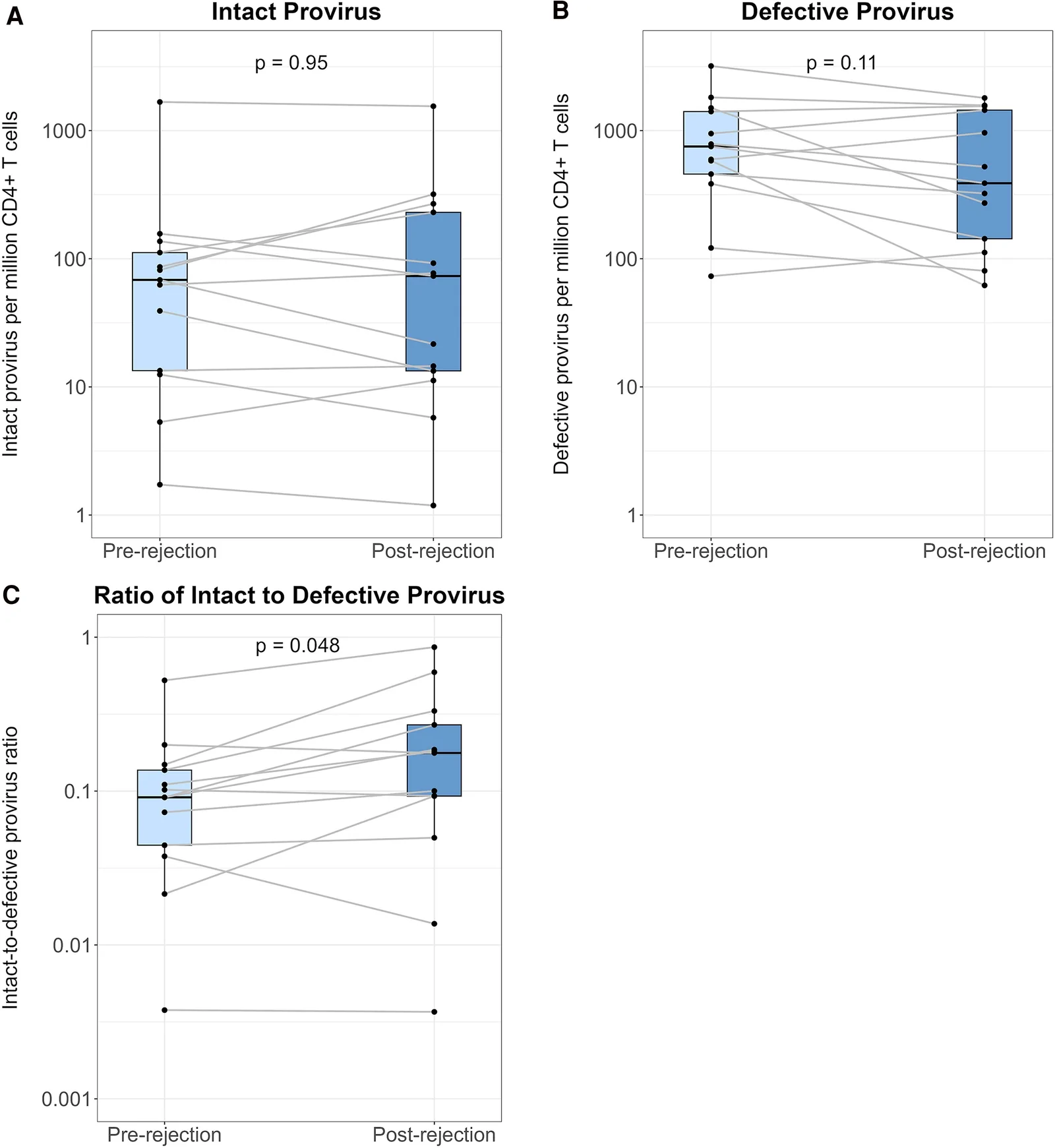
Intact and defective provirus frequencies per million CD4+ T-cells stratified by pre- and post-rejection in the kidney rejection cohort. *A*, Intact (*B*) defective provirus per million CD4+ T cells (*C*) Intact to defective proviral ratios. Count per million CD4+ T cells is plotted on the y-axis using a log10 scale. Pre- and post-rejection visits correspond to the last scheduled visit before rejection and the scheduled visit at or following rejection, with scheduled visits at weeks 0, 13, 26, and 52. Wilcoxon signed-rank tests were used to calculate *P* values comparing pre- and post-rejection measurements among participants with rejection. Among 14 recipients with kidney organ rejection, one was omitted from analysis due to missing provirus data.

There were no significant changes in intact or defective provirus among participants pre- versus post-rejection (Figure 2*A* and 2*B*; Supplementary Figure 1). However, there was a significant increase in the ratio of intact/defective provirus (*P* & .048). For the participants in this cohort, the counts of intact provirus ranged from a median of 68.38 copies/106 cells (IQR = 13.38–111.69) during the pre-rejection phase to 73.28 copies/106 cells (IQR = 13.33–230.15) post-rejection. Defective provirus ranged from a median of 751.53 copies/106 cells (IQR = 458.10–1407.57) during the pre-rejection phase to 388.55 copies/106 cells (IQR = 143.20–1443.81) post-rejection. The ratio of intact/defective provirus ranged from a median of 0.09 copies/106 cells (IQR = 0.04–0.14) pre-rejection to 0.18 copies/106 cells (IQR = 0.09–0.27) post-rejection.

## DISCUSSION

The LVR presents a major challenge to HIV curative therapy. The LVR is primarily made up of a population of HIV+ resting CD4+ T cells, which can re-activate and restore viremia if ART is discontinued. Our longitudinal results demonstrate that in PWH receiving allografts from donors with or without HIV there was no significant difference in the size of the intact or defective proviruses by rejection status and no changes in the LVR post-rejection. These findings support the feasibility of HIV+ to HIV+ organ transplantation, and that the LVR is stable in transplant recipients with HIV.

Our study findings suggest that, rather than the LVR, alternative factors such as chronic inflammation, immune dysregulation (expansion of allosensitized memory T cells) and dysregulation of immunosuppression due to ART drug interactions reducing calcineurin levels may play a greater role in kidney allograft rejection. As such, immunosuppressive and induction therapy regimens that target lymphocytes are important. This was supported by previous HOPE in Action data that showed that recipients who received lymphocyte-depleting induction were found to have reduced risk of rejection^6^. Those findings are in-line with Scientific Registry of Transplant Recipients reports, which showed in two separate analyses of HIV+ kidney transplant recipients, that the relative risk of acute rejection rate was substantially reduced in those who received ATG induction therapy [13, 14].

Regarding LVR stability in D+ organ transplant recipients with HIV, our current findings in the kidney transplantation recipients with HIV are consistent with prior findings focused on liver transplant recipients. These data demonstrated LVR stability post-transplant (comparing the LVR from time of transplant up to 52 weeks post-transplant) with CD4+ T-cell counts, intact and defective proviruses all comparable to baseline levels and no differences observed among recipients of livers from HIV+ or HIV− donors [12]. Importantly, these findings suggested that the latently infected cell population can be maintained even with the transplant of the liver, a large secondary lymphoid organ which contains substantial residing CD4+ T cells and tissue resident macrophages. The current study built upon these foundational studies to demonstrate within the kidney transplant cohort of the HOPE multicenter cohort, that in recipients who experienced rejection, there was no concurrent alteration within the LVR.

Importantly, there were limitations with our study. Our study used CD4+ T cells from the peripheral circulation to determine the LVR. It is possible that cellular populations residing in the kidney (kidney-residing CD4+ T cells or macrophages) may have alterations in the LVR that would have gone undetected in our analysis. We did not assess cell-associated HIV RNA or host transcriptomics; thus we cannot evaluate whether transient HIV transcription or inflammatory gene expression signatures associate with rejection. Additionally, the participants in this study were on different forms of induction and chronic immunosuppressive therapy, a proven method to reduce the rate of organ rejection. However, these therapies also deplete the T-cell population, which may have affected our results [3]. Finally, our study followed recipients only after transplant with either a D+ or D− kidney for up to 52 weeks, a significant longitudinal study. However, reports have shown that the LVR can re-establish viral load even 20 years post-HIV infection. Therefore, studies with multiyear follow-up are needed to provide critical data on long-term longitudinal changes in the viral reservoir among PLWH.

Our findings support that the LVR is not a major factor associated with the risk of short-term organ rejection, and reaffirm the recent U.S. Department of Health and Human Services removal of clinical research requirements for transplantation of kidneys and livers from donors with HIV to expand access to life-saving organ transplantation for PWH [15].

## Supplementary Material

jiag154_Supplementary_Data

## Supplementary Data

Supplementary materials are available at *The Journal of Infectious Diseases* online (http://jid.oxfordjournals.org/). Supplementary materials consist of data provided by the author that are published to benefit the reader. The posted materials are not copyedited. The contents of all supplementary data are the sole responsibility of the authors. Questions or messages regarding errors should be addressed to the author.

## References

[1] Chaillon A, Gianella S, Dellicour S, et al HIV persists throughout deep tissues with repopulation from multiple anatomical sources. J Clin Invest 2020; 130:1699–712. 31910162 10.1172/JCI134815 PMC7108926

[2] Gaebler C, Kor S, Allers K, et al Sustained HIV-1 remission after heterozygous CCR5Δ32 stem cell transplantation. Nature 2025; 650:701–9. 41326734 10.1038/s41586-025-09893-0 PMC12916306

[3] Reeves DB, Litchford M, Fish CS, et al Intact HIV DNA decays in children with and without complete viral load suppression. PLoS Pathog. 2025; 21:e1013003. 40184428 10.1371/journal.ppat.1013003 PMC12002518

[4] McMyn NF, Varriale J, Fray EJ, et al The latent reservoir of inducible, infectious HIV-1 does not decrease despite decades of antiretroviral therapy. J Clin Invest 2023; 133:e171554. 37463049 10.1172/JCI171554 PMC10471168

[5] Stock PG, Barin B, Murphy B, et al Outcomes of kidney transplantation in HIV-infected recipients. N Engl J Med 2010; 363:2004–14. 21083386 10.1056/NEJMoa1001197 PMC3028983

[6] Durand CM, Zhang W, Brown DM, et al A prospective multicenter pilot study of HIV-positive deceased donor to HIV-positive recipient kidney transplantation: HOPE in action. Am J Transplant 2021; 21:1754–64. 32701209 10.1111/ajt.16205 PMC8073960

[7] Durand CM, Massie A, Florman S, et al Safety of kidney transplantation from donors with HIV. N Engl J Med 2024; 391:1390–401. 39413376 10.1056/NEJMoa2403733 PMC11606542

[8] Lee TH, Wegermann K, Sutha K, et al Beyond the HIV Organ Policy Equity Act: roadmap to expanding kidney and liver transplants for people with human immunodeficiency virus utilizing grafts from donors with human immunodeficiency virus. Am J Transplant 2025; 25:2057–66. 40368062 10.1016/j.ajt.2025.05.013

[9] Bonny TS, Kirby C, Martens C, et al Outcomes of donor-derived superinfection screening in HIV-positive to HIV-positive kidney and liver transplantation: a multicentre, prospective, observational study. Lancet HIV 2020; 7:e611–9. 32730756 10.1016/S2352-3018(20)30200-9 PMC8073978

[10] Bruner KM, Wang Z, Simonetti FR, et al A quantitative approach for measuring the reservoir of latent HIV-1 proviruses. Nature 2019; 566:120–5. 30700913 10.1038/s41586-019-0898-8 PMC6447073

[11] Benner SE, Eby Y, Zhu X, et al The effect of induction immunosuppression for kidney transplant on the latent HIV reservoir. JCI Insight 2022; 7:e162968. 36345940 10.1172/jci.insight.162968 PMC9675561

[12] Benner SE, Zhu X, Hussain S, et al HIV-Positive Liver transplant does not Alter the latent viral reservoir in recipients with antiretroviral therapy-suppressed HIV. J Infect Dis 2023; 228:1274–9. 37379584 10.1093/infdis/jiad241 PMC10629701

[13] Kucirka LM, Durand CM, Bae S, et al Induction immunosuppression and clinical outcomes in kidney transplant recipients infected with human immunodeficiency virus. Am J Transplant 2016; 16:2368–76. 27111897 10.1111/ajt.13840 PMC4956509

[14] Locke JE, James NT, Mannon RB, et al Immunosuppression regimen and the risk of acute rejection in HIV-infected kidney transplant recipients. Transplantation 2014; 97:446–50. 24162248 10.1097/01.TP.0000436905.54640.8c

[15] Department of Health and Human Services Organ Procurement and Transplantation: Implementation of the HIV Organ Policy Equity (HOPE) Act. 2024. Available at: https://www.federalregister.gov/documents/2024/11/27/2024-27410/organ-procurement-and-transplantation-implementation-of-the-hiv-organ-policy-equity-hope-act. Accessed February 2026

