# Supplementary material for: Allograft Rejection and the Latent HIV Reservoir in Kidney Transplant Recipients With HIV
Source: J Infect Dis. 2026 Mar 13;234(2):e363–8. doi: 10.1093/infdis/jiag154 (PMC13046422; doi:10.1093/infdis/jiag154)
Supplement: Supplementary file 1 [file jiag154_supplementary_data.docx]

**Supplemental Table 1. Baseline Characteristics of Kidney Transplant Recipients, Stratified by Rejection Status**

| **Baseline characteristics** | **Recipients without kidney organ rejection**  **(n=55)** | **Recipients with kidney organ rejection**  **(n=14)** | ***p* value** |
| --- | --- | --- | --- |
| **Median age at time of transplant (IQR), years** | 54 (49-63) | 47 (36-53) | **0.006** |
| **Male sex, n (%)** | 40 (72.7%) | 12 (85.7%) | 0.314 |
| **Race, n (%)** |  |  | 0.285 |
| White | 11 (20.0%) | 2 (14.3%) |  |
| Black | 37 (67.3%) | 12 (85.7%) |  |
| Other | 7 (12.7%) | 0 (0.0%) |  |
| **Cytomegalovirus IgG Ab positive, n (%)** | 53 (96.4%) | 13 (92.9%) | 0.566 |
| **Hepatitis B IgG or total core Ab positive, n (%)** | 23 (41.8%) | 5 (35.7%) | 0.678 |
| **Hepatitis C antibody positive, n (%)** | 8 (14.5%) | 1 (7.1%) | 0.463 |
| **Median CD4+ T cell count (IQR), cells/µL** | 498 (382-736) | 558 (321-755) | 0.560 |
| **HIV RNA < 200 copies/mL, n (%)** | 54 (98.2%) | 14 (100.0%) | 0.611 |
| **Kidney donor with HIV, n (%)** | 33 (60.0%) | 7 (50.0%) | 0.499 |
| **Antiretroviral therapy, n (%)** |  |  |  |
| Containing INSTI | 54 (98.2%) | 13 (92.9%) | 0.289 |
| Containing NNRTI | 19 (34.5%) | 4 (28.6%) | 0.672 |
| Containing protease or cobicistat | 4 (7.3%) | 1 (7.1%) | 0.987 |
| **Cause of kidney failure** |  |  | 0.473 |
| HIV-associated nephropathy | 19 (34.5%) | 7 (50.0%) |  |
| Diabetes | 12 (21.8%) | 1 (7.1%) |  |
| Hypertension | 12 (21.8%) | 2 (14.3%) |  |
| Other | 12 (21.8%) | 4 (28.6%) |  |
| **Median duration of HIV (IQR), years** | 24 (16-32) | 18 (14-24) | 0.092 |
| **Induction immunosuppression, n (%)** |  |  | 0.230 |
| ATG or ATGAM | 29 (53.7%) | 5 (35.7%) |  |
| Basiliximab | 25 (46.3%) | 9 (64.3%) |  |
| **Maintenance immunosuppression, n(%)** |  |  |  |
| Tacrolimus | 52 (94.5%) | 14 (100.0%) | 0.372 |
| MMF or MFA | 52 (94.5%) | 14 (100.0%) | 0.372 |

Note: Categorical variables are presented as n (%); continuous variables are presented as median (IQR). Racial information for those described as “other” was collected from participant records and includes Asian, American Indian or Alaska Native, Native Hawaiian or Other Pacific Islander, Puerto Rican, as well as nonspecific, not reported, and not documented identities. Antiretroviral therapies are not mutually exclusive. Causes of kidney failure are not mutually exclusive; individual patients may have more than one contributing etiology. The Wilcoxon rank-sum test (Mann–Whitney U test) was used for continuous variables, and Fisher's exact test was used for categorical variables, comparing recipients with and without kidney organ rejection. Column percentages may not sum to 100% due to rounding.

Abbreviation: INSTI, integrase strand transfer inhibitor; NNRTI, non-nucleoside reverse transcriptase inhibitor; ATG, anti-thymocyte globulin; ATGAM (equine anti-thymocyte globulin); MMF, mycophenolate mofetil; MFA, mycophenolate acid.

**Supplemental Table 2. Characteristics of Kidney Rejection Episodes by Donor HIV Status**

| **Characteristics of rejection episodes** | **HIV D+/R+,**  **(N=8)** | **HIV D-/R+**  **(N=11)** | **Overall**  **(N=19)** |
| --- | --- | --- | --- |
| **Biopsy performed** | 6/8 | 9/11 | 15/19 |
| **Recipients with rejection** | 7 | 7 | 14 |
| 1 event | 6/7 | 4/7 | 10/14 |
| 2 events | 1/7 | 2/7 | 3/14 |
| 3 events | 0/7 | 1/7 | 1/14 |
| **Rejection type** |  |  |  |
| Cellular | 6/6 | 9/9 | 15/15 |
| Antibody mediated | 0/6 | 0/9 | 0/15 |
| **Types of treatment received** |  |  |  |
| Steroids only | 4/8 | 7/11 | 11/19 |
| IVIG + steroids | 1/8 | 1/11 | 2/19 |
| ATG + steroids | 0/8 | 1/11 | 1/19 |
| IVIG + ATG + steroids | 0/8 | 1/11 | 1/19 |
| IVIG + steroids + rituximab | 1/8 | 0/11 | 1/19 |
| No treatment | 2/8 | 1/11 | 3/19 |

Abbreviation: HIV D+/R+: Transplantation of kidneys from donors with HIV to recipients with HIV; HIV D-/R+: Transplantation of kidneys from donors without HIV to recipients with HIV; IVIG: Intravenous immunoglobulin; ATG: Anti-thymocyte globulin.

**Supplemental Figure 1. Individual-level changes in intact provirus, defective provirus, and the intact-to-defective ratio from baseline to week 52, stratified by rejection status**

Note: Each line represents an individual, and each dot represents a time point. Among 14 recipients with kidney organ rejection, one was omitted from analysis due to missing provirus data.
